# Multi-omics analysis unravels the underlying mechanisms of poor prognosis and differential therapeutic responses of solid predominant lung adenocarcinoma

**DOI:** 10.3389/fimmu.2023.1101649

**Published:** 2023-02-09

**Authors:** Feng Li, Shuaibo Wang, Yaru Wang, Zhuoheng Lv, Donghui Jin, Hang Yi, Li Fu, Suokai Zhai, Ting Xiao, Yousheng Mao

**Affiliations:** ^1^ Department of Thoracic Surgery, National Cancer Center/National Clinical Research Center for Cancer/Cancer Hospital, Chinese Academy of Medical Sciences and Peking Union Medical College, Beijing, China; ^2^ State Key Laboratory of Molecular Oncology, Department of Etiology and Carcinogenesis, National Cancer Center/National Clinical Research Center for Cancer/Cancer Hospital, Chinese Academy of Medical Sciences and Peking Union Medical College, Beijing, China; ^3^ Department of Cardiothoracic Surgery, Zibo First Hospital, Weifang Medical University, Zibo, Shandong, China

**Keywords:** solid, non-solid, lung adenocarcinoma, multi-omics, immunotherapy

## Abstract

**Background:**

Solid predominant adenocarcinoma (SPA) has been reported to be a subtype with poor prognosis and unsatisfactory response to chemotherapy and targeted therapy in lung adenocarcinoma (LUAD). However, the underlying mechanisms remain largely unknown and the suitability of immunotherapy for SPA has not been investigated.

**Methods:**

We conducted a multi-omics analysis of 1078 untreated LUAD patients with clinicopathologic, genomic, transcriptomic, and proteomic data from both public and internal cohorts to determine the underlying mechanisms of poor prognosis and differential therapeutic responses of SPA and to investigate the potential of immunotherapy for SPA. The suitability of immunotherapy for SPA was further confirmed in a cohort of LUAD patients who received neoadjuvant immunotherapy in our center.

**Results:**

Along with its aggressive clinicopathologic behaviors, SPA had significantly higher tumor mutation burden (TMB) and number of pathways altered, lower TTF-1 and Napsin-A expression, higher proliferation score and a more immunoresistant microenvironment than non-solid predominant adenocarcinoma (Non-SPA), accounting for its worse prognosis. Additionally, SPA had significantly lower frequency of therapeutically targetable driver mutations and higher frequency of EGFR/TP53 co-mutation which was related to resistance to EGFR tyrosine kinase inhibitors, indicating a lower potential for targeted therapy. Meanwhile, SPA was enriched for molecular features associated with poor response to chemotherapy (higher chemoresistence signature score, lower chemotherapy response signature score, hypoxic microenvironment, and higher frequency of TP53 mutation). Instead, muti-omics profiling revealed that SPA had stronger immunogenicity and was enriched for positive biomarkers for immunotherapy (higher TMB and T cell receptor diversity; higher PD-L1 expression and more immune cell infiltration; higher frequency of gene mutations predicting efficacious immunotherapy, and elevated expression of immunotherapy-related gene signatures). Furthermore, in the cohort of LUAD patients who received neoadjuvant immunotherapy, SPA had higher pathological regression rates than Non-SPA and patients with major pathological response were enriched in SPA, confirming that SPA was more prone to respond to immunotherapy.

**Conclusions:**

Compared with Non-SPA, SPA was enriched for molecular features associated with poor prognosis, unsatisfactory response to chemotherapy and targeted therapy, and good response to immunotherapy, indicating more suitability for immunotherapy while less suitability for chemotherapy and targeted therapy.

## Introduction

Given the significant heterogeneity in lung adenocarcinoma (LUAD), a new classification system for LUAD was proposed in 2011, which classified invasive LUAD into five subtypes based on the predominant histologic pattern, including lepidic, acinar, papillary, micropapillary and solid (1). This new subtype classification has been reported to be strongly related to prognosis and therapeutic vulnerabilities (2–5).

Solid predominant adenocarcinoma (SPA) has been reported to be a subtype with early recurrence and poor prognosis (2, 5) while the biological and molecular mechanisms are largely unknown. Caso et al. performed the first in-depth analysis of genomic landscape of LUAD histologic subtypes and showed that micropapillary or solid predominant adenocarcinoma had higher tumor mutational burden (TMB), increased chromosomal instability, and more oncogenic pathway alterations as compared with lepidic, acinar or papillary predominant adenocarcinoma (6). Zhang et al. compared the transcriptomic profiles between SPA and acinar predominant adenocarcinoma and showed that SPA was enriched in pathways associated with RNA polymerase activity and p53 inactivation (7). Zhou et al. performed comprehensive proteomic analyses of high-risk LUAD subtypes (micropapillary and solid) and low-risk subtype (lepidic) and found that differentially expressed proteins were enriched in pathways involved in remodeling of extracellular matrix and activation of DNA replication and cell cycle (8). Dong et al. investigated the immune profiles of LUAD histologic subtypes and discovered that SPA was correlated with an immunoresistant tumor microenvironment (3). Even though these studies provided new perspectives into the mechanisms underlying the poor prognosis of SPA, they were largely limited by the small sample size and single-dimensional analysis. A comprehensive investigation of multiple-dimensional data from larger LUAD cohorts is urgently needed.

Like other subtypes of LUAD, before the approval of immunotherapy, cytotoxic chemotherapy was the mainstay of treatment for SPA without driver mutations, while for those with driver gene aberrations, such as EGFR mutation, targeted therapy is a standard therapy (9). Previous studies showed that the efficacy of adjuvant chemotherapy was not satisfactory in SPA and SPA was significantly associated with poor response to EGFR tyrosine kinase inhibitors (TKI) in patients with EGFR-activating mutations (3–5). However, the molecular mechanisms responsible for the poor response to chemotherapy and targeted therapy in SPA remain unknown.

Immunotherapy have revolutionized the treatment of lung cancer, bringing unprecedented prolongation of life. Despite this, durable response only occurs in a tiny minority, necessitating the identification of patients who may benefit from it (10, 11). Given the unsatisfactory response to chemotherapy and targeted therapy in SPA, the suitability and efficacy of immunotherapy for SPA need to be thoroughly investigated. Here, we conducted a comprehensive analysis of multiple-dimensional data including clinical, genomic, transcriptomic, and proteomic data from both public and internal cohorts to determine the underlying mechanisms of poor prognosis and differential therapeutic responses of SPA as well as the potential of immunotherapy for SPA.

## Materials and methods

### LUAD data sets

Four cohorts were included for evaluating the clinicopathologic, genomic, transcriptomic, and immune profiles of SPA: ① LUAD-TCGA: 199 untreated LUAD patients with genomic data (197 with transcriptomic data, 148 with proteomic data) and confirmed histologic subtypes from The Cancer Genome Atlas (TCGA) repository (12); ② LUAD-MSKCC: 604 untreated LUAD patients with next-generation sequencing data and definite histologic subtypes from the MSKCC cohort (6); ③ LUAD-Singapore: 173 untreated LUAD patients with detailed histologic subtypes (172 with genomic data, 141 with transcriptomic data) from Singapore (13); ④ LUAD-NCC: 437 untreated LUAD patients (103 with genomic and proteomic data, 51 with transcriptomic data) from our center (14). LUAD histologic subgroups were classified into 2 categories: SPA versus Non-solid predominant adenocarcinoma (Non-SPA).

**Figure 1:**
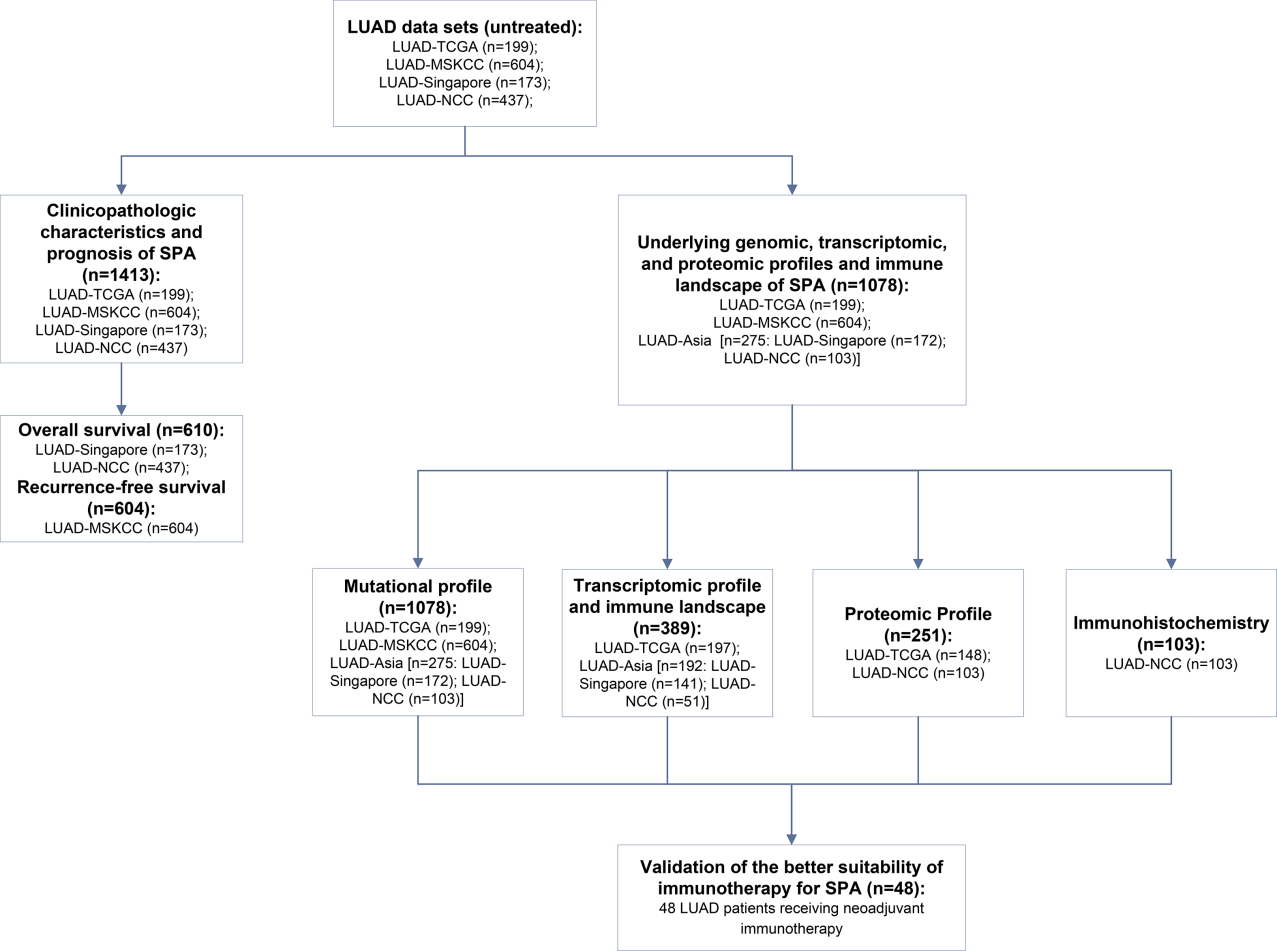
Flow diagram illustrating the study design.

In addition, a cohort of 48 LUAD patients who received neoadjuvant immunotherapy in our center was used to validate the better suitability of immunotherapy for SPA. A flow diagram was drawn to illustrate the study design (**Figure 1**). The baseline information of the main data sets included is summarized in **Table S1**. This study was approved by the Ethics Committee of National Cancer Center/Cancer Hospital, Chinese Academy of Medical Sciences and Peking Union Medical College (Approval No. 2016YJC-01). Written informed consent was obtained from all participants.

### Collection of clinical and multi-omics data

Whole exome sequencing and RNA sequencing were performed in LUAD-TCGA, LUAD-Singapore and LUAD-NCC (12–14). MSK-IMPACT next-generation sequencing was performed in LUAD-MSKCC (6). The protein quantitation was based on reverse phase protein array (RPPA) in LUAD-TCGA and mass spectrometry in LUAD-NCC (12, 14). The detailed methods and experimental procedures regarding DNA, RNA, and protein extraction from tumors, library preparation, sequencing, quality control, and subsequent data processing have been previously reported (6, 12–14). The updated clinical, genomic, transcriptomic and proteomic data of LUAD-TCGA was retrieved from the TCGA data portal (https://gdc.cancer.gov/) using the R package “TCGAbiolinks” (15) and the corresponding histologic subtypes of patients were obtained from the supplementary material of the study conducted by TCGA Research Network (12). The clinical and genomic data of LUAD-MSKCC was obtained from the cBioPortal database (https://www.cbioportal.org/study/summary?id=luad_mskcc_2020). The clinical, genomic, and transcriptomic data of LUAD-Singapore was derived from the Singapore Oncology Data Portal (https://src.gisapps.org/OncoSG/) and the histologic subtype of cases were obtained from the supplementary material of the previously published study (13). The genomic, transcriptomic and proteomic data of LUAD-NCC was collected from the supplementary material of our previously published research (14). The genomic and transcriptomic data of LUAD-Singapore and LUAD-NCC were integrated into a combined cohort (LUAD-Asia) due to similar ethnic backgrounds. For rare mutational events, LUAD-TCGA, LUAD-MSKCC, and LUAD-Asia were combined into a larger cohort (LUAD-Combined).

### Genomic profiling

The mutation status of each gene was inferred from the mutation MAF files by the maftools package (16). OncoPrints constructed by the ComplexHeatmap R package (17) were used to depict the mutation landscape. TMB was defined as the total nonsynonymous somatic mutation counts in coding regions (megabases). Neoantigen load, T cell receptor diversity, intratumor heterogeneity scores and homologous recombination deficiency scores of patients in LUAD-TCGA were retrieved from the supplementary material of a previous research (18). Deleterious NOTCH mutations were determined by PolyPhen-2 (http://genetics.bwh.harvard.edu/pph2/), which could classify a given mutation as damaging or benign (19, 20). The deconstructSigs R package (21) with default parameters was used to derive COSMIC mutational signatures (22), which account for the observed mutational profile in each patient. Therapeutically targetable alterations were annotated using OncoKB (https://www.oncokb.org/) (23), which collects information from the published research and defines drug actionability according to the clinical evidence.

### Molecular expression and subtype assignments

The gene expression levels were measured as fragments per kilobase per million mapped reads and were then log2 transformed. For combination and comparison of expression data from different datasets, the expression level of each gene was further normalized by a z-score with mean value = 0 and standard deviation = 1. Heatmap was used to depict the gene expression levels by the pheatmap R package. Protein quantitation was evaluated by a label-free quantification algorithm (iBAQ) in LUAD-NCC and values of iBAQ were log2 transformed if necessary. Protein quantitation in LUAD-TCGA was measured by RPPA. The TCGA molecular subtypes were determined using the published LUAD 506-gene nearest centroid classifier (24). The TCGA immune subtypes and the Karolinska proteomic subtypes were obtained from previously published studies (18, 25).

### Pathway enrichment analysis and signature score estimation

Unbiased gene set enrichment analysis (GSEA) was performed to identify pathways enriched in SPA and Non-SPA using the javaGSEA Desktop Application (26). HALLMARK gene sets from the MSigDB database (26) were selected for GSEA. The threshold was set at false discovery rate (FDR) <0.1. The enrichment score in GSEA was calculated by first ranking the genes from the most to least significant with respect to the two phenotypes (SPA versus Non-SPA), the entire ranked list was then used to assess how the genes of each gene set were distributed across the ranked list. Functional gene expression signatures manually curated (18, 27–32) were used to investigate the correlation between pathological subtypes and other relevant biological processes (**Table S2**). For each signature, we performed gene set variation analysis and assigned a signature score to each patient using the GSVA R package (33). The 18-gene T cell-inflamed gene expression profile (GEP) score was calculated as weighted sum of the normalized expression values of the genes (27) and the weightings for each gene in the signature (**Table S3**) were obtained from the published patent (34).

### Immune cell infiltration estimation

The xCell tool (https://xcell.ucsf.edu) was used to analyze the relative levels of infiltration of 64 immune and non-immune cell types in the tumor immune microenvironment from the RNA sequencing data (35). This method combined the advantages of gene enrichment analysis and deconvolution approaches to evaluate the enrichment of immune cells. The spatial fractions of tumor regions with infiltrating immune cells estimated by analysis of the mapped TCGA digitized hematoxylin-eosin (H&E)-stained slides were obtained from a published study (36).

### Immunohistochemistry

A total of 103 LUAD patients from LUAD-NCC were assessed by immunohistochemistry (IHC) staining with PD-L1 (clone SP263; Roche Ventana), CD4 (ZA-0519; Zsbio Tech), CD8 (ZA-0508; Zsbio Tech), CD19 (ZM-0038; Zsbio Tech), CD68 (ZM-0060; Zsbio Tech) and CD163 (ZM-0428; Zsbio Tech) antibodies. Formalin-fixed, paraffin-embedded LUAD specimens were cut into 4 mm slides for IHC staining. Slides were stained using an automated Leica Bond staining system according to the manufacturer’s protocol. PD-L1 expression scores were defined as the percentage of tumor cells with membranous staining. Staining status was stratified into three subgroups: negative (PD-L1 <1%), intermediate (PD-L1 1-49%), and high (PD-L1 ≧50%). For CD4 and CD8, the proportion of positive cells was assessed as low density (≦25%), intermediate density (25-50%), and high density (> 50%). For CD19, CD68 and CD163, the percentage of stained cells was classified as follows: low density (≦10%), intermediate density (10-20%), and high density (> 20%). All slides were evaluated by two pathologists and any disagreement was resolved by consensus using a multi-head microscope.

### Statistical analyses

Wilcoxon rank-sum test (or Kruskal-Wallis test) and χ2 test (or fisher’s exact test) were used to assess differences between continuous and categorical variables, respectively. Survival analysis was conducted using the Kaplan–Meier method and log-rank test. Hazard ratios (HRs) were determined by Cox regression analyses. Forest plots showing HRs and confidence intervals were drawn. All statistical analyses were performed with the STATA 16.0 and R 3.6.3. All reported P values were two-sided, and P <0.05 was considered statistically significant unless otherwise specified. For multiple testing, P-values were adjusted using Benjamini–Hochberg FDR correction and FDR q value < 0.10 was considered significant.

## Results

### Clinicopathologic characteristics and prognosis of SPA and Non-SPA

**Figure 2:**
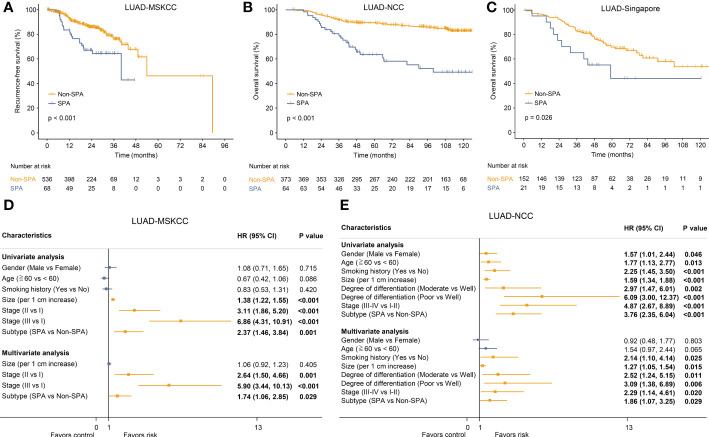
Survival analyses of SPA versus Non-SPA. **(A)** Recurrence-free survival of SPA and Non-SPA in LUAD-MSKCC. **(B)** Overall survival of SPA and Non-SPA in LUAD-NCC. **(C)** Overall survival of SPA and Non-SPA in LUAD-Singapore. **(D, E)** Univariate and multivariate Cox regression analyses of the pathological subtype and other clinicopathologic features regarding prognostic value in LUAD-MSKCC and LUAD-NCC. SPA, solid predominant adenocarcinoma; Non-SPA, non-solid predominant adenocarcinoma; CI, confidence interval.

The differences in clinicopathologic characteristics between SPA and Non-SPA are detailed in **Table S4**. SPA had distinct clinicopathologic characteristics as compared with Non-SPA. SPA was more common in patients with a history of smoking and tended to have a higher rate of lymph node metastasis, a higher pathological stage and a lower degree of differentiation. Next, the survival of SPA and Non-SPA was compared. SPA showed significantly shorter recurrence-free survival (P<0.001; LUAD-MSKCC) and overall survival (P<0.001; LUAD-NCC) than did Non-SPA (**Figures 2A, B**). Multivariate cox regression analyses confirmed that SPA was an unfavorable prognostic factor for both recurrence-free survival (HR: 1.74, P=0.029; **Figure 2D**) and overall survival (HR: 1.86, P=0.029; **Figure 2E**). In LUAD-Singapore, we also observed a worse overall survival in SPA (P=0.026; **Figure 2C**). To uncover the underlying mechanisms, we performed a multi-omics analysis.

### Genomic profiles and immunotherapy-related genetic mutations in SPA and Non-SPA

**Figure 3:**
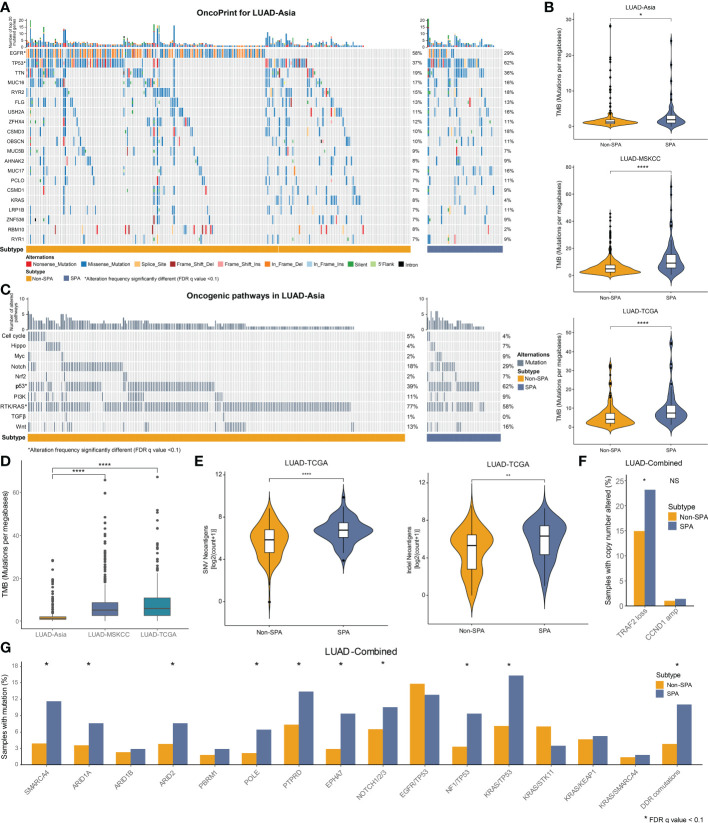
Mutational landscape of SPA and Non-SPA. **(A)** OncoPrint of the top 20 mutated genes in LUAD-Asia. *FDR q value < 0.1 **(B)** TMB of SPA and Non-SPA in the three cohorts. ****P < 0.0001, *P < 0.05 **(C)** OncoPrint of the 10 hallmark oncogenic pathways in LUAD-Asia. *FDR q value < 0.1 **(D)** Differences in TMB between races. ****P < 0.0001 **(E)** Comparison of neoantigen load between SPA and Non-SPA in LUAD-TCGA. ****P < 0.0001, **P < 0.01 **(F)** Comparison of TRAF2 loss and CCND1 amplification between SPA and Non-SPA in LUAD-Combined. *P < 0.05; NS, not significant **(G)** Frequency of gene alterations associated with response to immunotherapy and co-mutations in SPA as compared with Non-SPA in the combined cohort (LUAD-Combined). *FDR q value < 0.1. SPA, solid predominant adenocarcinoma; Non-SPA, non-solid predominant adenocarcinoma; TMB, tumor mutational burden; DDR, DNA damage response.

We first depicted comprehensive genomic landscapes of SPA and Non-SPA in LUAD-Asia, LUAD-MSKCC and LUAD-TCGA (**Figures 3A**, **S1A, C**). Despite the genetic diversity of cancer genome between races, SPA and Non-SPA showed distinct genomic profiles across the three cohorts. Consistently higher levels of TMB was observed in SPA (**Figure 3B**) although the median TMB differed markedly between races (**Figure 3D**). Similarly, SPA had higher neoantigen load than Non-SPA (**Figure 3E**), indicating potential suitability for immunotherapy.

Next, we compared the mutation rates of the curated genes (19, 37–42) which were associated with response to immunotherapy between SPA and Non-SPA. Three cohorts were merged into a cohort (LUAD-Combined) due to the low mutation rates of these genes. The results showed that SPA had significantly higher frequency of SMRCA4, ARID1A, ARID2, POLE, PTPRD, and EPHA7 mutations (**Figure 3G**). In terms of co-occurring mutations (12, 38, 43–45), NF1/TP53 co-mutation, KRAS/TP53 co-mutation, and co-mutations in DNA damage response (DDR) pathways were enriched in SPA. A recent study demonstrated that TRAF2 loss and CCND1 amplification were associated with response and resistance to immunotherapy, respectively (46). Here, we found that TRAF2 loss was significantly enriched in SPA while no difference was found in CCND1 amplification between SPA and Non-SPA (**Figure 3F**).

### Driver mutations and therapeutically targetable mutations in SPA and Non-SPA

**Figure 4:**
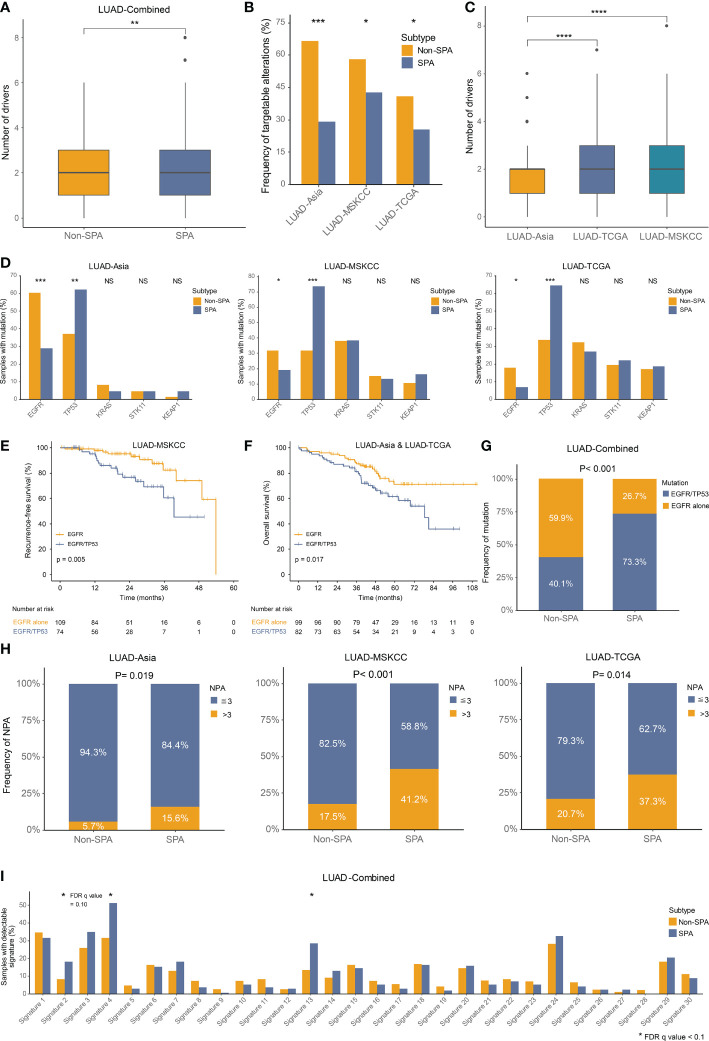
Driver mutations, oncogenic pathways and somatic mutational signatures in SPA and Non-SPA **(A)** Number of driver mutations in SPA and Non-SPA. **P < 0.01 **(B)** Frequency of targetable alterations in SPA and Non-SPA across the three cohorts. ***P < 0.001, *P < 0.05 **(C)** Differences in number of driver mutations between races. ****P < 0.0001 **(D)** Mutation frequency of EGFR, TP53, KRAS, STK11, and KEAP1 in SPA and Non-SPA across the three cohorts. ***P < 0.001, **P < 0.01, *P < 0.05; NS, not significant **(E)** Recurrence-free survival of EGFR mutation alone versus EGFR/TP53 co-mutation. **(F)** Overall survival of EGFR mutation versus EGFR/TP53 co-mutation. **(G)** Frequency of EGFR mutation alone and EGFR/TP53 co-mutation in SPA and Non-SPA. **(H)** Frequency of three or more pathways altered in SPA and Non-SPA across the three cohorts. **(I)** Frequency of 30 COSMIC signatures in SPA and Non-SPA. *FDR q value < 0.1. SPA, solid predominant adenocarcinoma; Non-SPA, non-solid predominant adenocarcinoma; NPA, number of pathways altered.

We investigated the alteration frequencies of known driver mutations and those amenable to targeted therapies. Even though SPA had higher number of driver mutations as compared with Non-SPA (mean: 2.27 versus 1.91, P=0.002; **Figure 4A**), lower frequencies of therapeutically targetable driver alterations were observed in SPA across the three cohorts (**Figure 4B**). Similar to TMB, higher number of driver mutations was seen in LUAD-TCGA and LUAD-MSKCC as compared with LUAD-Asia (**Figure 4C**).

Next, we compared the mutation rates of five of the most common driver mutations in LUAD, including EGFR, TP53, KRAS, STK11, and KEAP1, which were reported to be correlated with tumor antigenicity and immunogenicity (43, 45, 47, 48). Two genes (TP53, EGFR) were found to be statistically significantly altered in SPA, as compared with Non-SPA across the three cohorts, with significantly higher frequency of TP53 and lower frequency of EGFR mutation in SPA (**Figure 4D**). Our group previously revealed that LUAD patients with EGFR/TP53 co-mutation had poorer overall survival than those with EGFR mutation alone in a small population (14). Here, with larger cohorts, we confirmed that the co-occurrence of EGFR and TP53 mutation was associated with earlier recurrence (P=0.005; **Figure 4E**) and poorer survival (P=0.017; **Figure 4F**) in EGFR-mutated patients. Importantly, in EGFR-mutated patients, we found that EGFR/TP53 co-mutation was enriched in SPA (P<0.001; **Figure 4G**).

### Oncogenic pathway alternations and somatic mutational signatures in SPA and Non-SPA

Finally, we evaluated the alteration frequencies of 10 hallmark oncogenic pathways (49) and 30 somatic mutational signatures (22) in SPA and Non-SPA. Six pathways were statistically significantly altered in SPA as compared with Non-SPA: Cell cycle, Hippo, Myc, Notch, p53, and PI3K (**Figures S1F**, **3C**, **S1B, D**). SPA had significantly higher number of pathways altered (NPA) than did Non-SPA (P<0.0001; **Figure S1E**). Tumors with three or more NPA were enriched in SPA across the three cohorts (**Figure 4H**). Mutational signature analysis revealed that the frequencies of APOBEC signature (signature 2 and 13) and smoking signature (signature 4) were significantly higher in SPA than in Non-SPA (**Figure 4I**). Meanwhile, a notable increase in the rate of transversion/transition was seen in SPA (**Figure S1G**). In addition, SPA showed higher level of homologous recombination deficiency signature (signature 3) with a marginal statistical difference (FDR q value = 0.10). Consistently, higher homologous recombination deficiency score was observed in SPA (**Figure S1H**).

### Expression of LUAD histologic markers and pathway enrichment in SPA versus Non-SPA

TTF-1 and Napsin-A are two canonical histologic markers used to demonstrate LUAD differentiation (1). In this study, consistent with the clinicopathologic characteristics of SPA (lower degree of differentiation and poorer prognosis), both the mRNA expression and protein abundance of these two markers showed significantly lower levels in SPA (**Figures S2A, B**), indicating that SPA might be atypical LUAD.

Unbiased GSEA of SPA versus Non-SPA showed that SPA was enriched in hallmark pathways associated with proliferation (E2F targets, G2M checkpoint, mTORC1 signaling, Mitotic spindle, MYC targets V1 and V2), immune response (Inflammatory response, IL-6 JAK STAT3 signaling, IFN-γ response, complement, allograft rejection, and IL-2 STAT5 signaling) and hypoxia (**Figure S2C**, **Table S5**), consistent with the recent finding that proliferation and immune axes segregated LUAD transcriptomic subgroups (13). However, Non-SPA was not enriched in any biological processes.

### Validation of the results of gene set enrichment analysis

To validate the results of GSEA, several functional gene expression signatures were curated (**Table S2**) and individual signature scores were calculated for each signature. Using the proliferation-related gene signature (18), we confirmed that SPA was more aggressive with higher proliferation scores (**Figure S2D**), which was further supported by the increased Ki67 expression (both mRNA and protein level) in SPA (**Figure S2E**). In terms of hypoxia, SPA displayed higher hypoxia scores and increased HIF-1α expression (**Figures S2F, G**), indicating a potential hypoxic microenvironment in this subtype.

**Figure 5:**
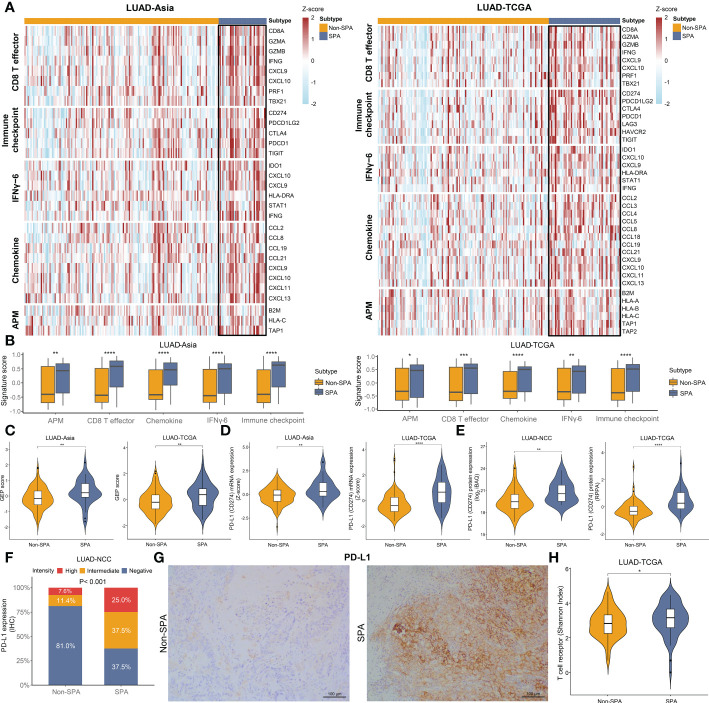
Immunity markers in SPA and Non-SPA. **(A)** Heatmaps of gene signatures associated with response to immunotherapy in LUAD-Asia and LUAD-TCGA. **(B)** Comparison of signature scores of the selected signatures between SPA and Non-SPA. **(C)** T cell-inflamed GEP scores in SPA and Non-SPA. **(D, E)** Comparison of mRNA expression and protein abundance of PD-L1 between SPA and Non-SPA. **(F)** Comparison of PD-L1 expression determined by immunohistochemistry between SPA and Non-SPA in LUAD-NCC. **(G)** Representative images of PD-L1 immunostaining in SPA and Non-SPA form LUAD-NCC. **(H)** Comparison of T cell receptor diversity between SPA and Non-SPA in LUAD-TCGA. SPA, solid predominant adenocarcinoma; Non-SPA, nonsolid predominant adenocarcinoma; CD8 T effector, effector CD8 T cell signature; IFNγ-6, six-gene IFNγ signature; APM, antigen presenting machinery; GEP, gene expression profile; RPPA, reverse phase protein array; IHC, immunohistochemistry. ****P < 0.0001, ***P < 0.001, **P < 0.01, *P < 0.05.

Since SPA showed a close relationship with immune-related pathways in GSEA, we explored the expression of several clusters of metagenes previously reported to be associated with response to immunotherapy (27, 31, 32), including an effector CD8 T cell signature (CD8 T effector), an immune checkpoint signature, a six-gene IFNγ signature (IFNγ-6), a chemokine signature and antigen presenting machinery (APM) signature. Heatmaps depicting the expression levels of these gene signatures revealed clear differences between SPA and Non-SPA. Specifically, SPA demonstrated higher levels of mRNA expression in these immunity markers (**Figure 5A**). To reinforce the robustness of our findings, scores of each signature were compared between these two subtypes. The results showed significantly higher scores across all these signatures in SPA, indicating preexisting immunity within this subtype (**Figure 5B**). Consistently, we observed significantly higher GEP scores (a pan-cancer predictor of response to immunotherapy (27)) in SPA (**Figure 5C**). Individual immune gene markers were also investigated. Both the LUAD-TCGA and LUAD-Asia cohorts showed significantly higher PD-L1 mRNA and protein expression in SPA than in Non-SPA (**Figures 5D, E**). IHC analysis confirmed that SPA had higher PD-L1 expression in the LUAD-NCC cohort (P<0.001, **Figures 5F, G**). In addition, SPA showed greater T cell receptor diversity (**Figure 5H**) and higher CXCL9/CXCL13 expression (**Figure S2H**).

### Distinct tumor immune microenvironment in SPA and Non-SPA

**Figure 6:**
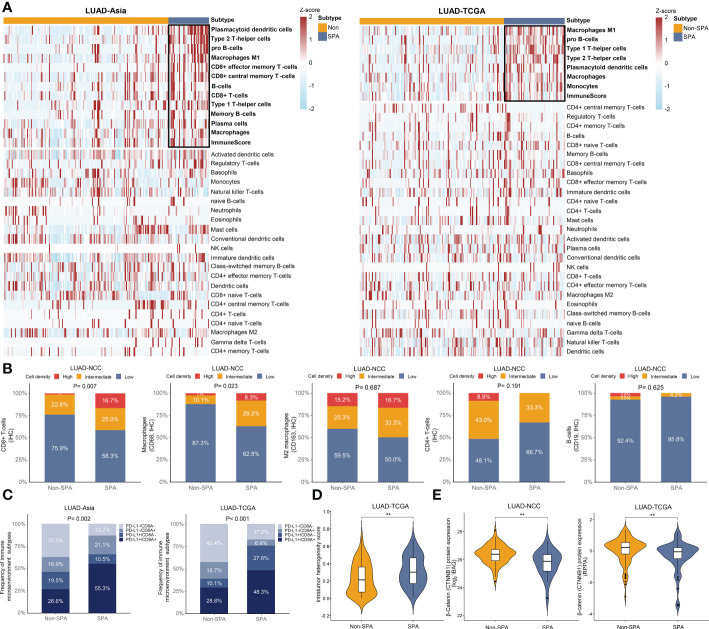
Tumor immune microenvironment of SPA and Non-SPA. **(A)** Tumor immune microenvironment of SPA and Non-SPA. **(B)** Immunohistochemistry evaluation of CD8+ T-cells, macrophages (CD68), M2 macrophages (CD163), CD4+ T-cells and B-cells (CD19) in SPA and Non-SPA in LUAD-NCC. **(C)** Correlation between pathological subtypes (SPA versus Non-SPA) and the four types of tumor immune microenvironment classified based on PD-L1 and CD8A expression. **(D)** Comparison of intra-tumor heterogeneity scores between SPA and Non-SPA in LUAD-TCGA. **(E)** Comparison of β-Catenin protein levels between SPA and Non-SPA in LUAD-NCC and LUAD-TCGA. SPA, solid predominant adenocarcinoma; Non-SPA, non-solid predominant adenocarcinoma; IHC, immunohistochemistry; RPPA, reverse phase protein array. **P < 0.01.

Next, we evaluated the infiltrating immune cells in SPA and Non-SPA. SPA had a distinct tumor immune microenvironment as compared with Non-SPA, with higher infiltration of M1 macrophages, Th1 and Th2 cells, CD8+ T cells and macrophages as well as higher immune scores in SPA (**Figures 6A**, **S3A**). No difference in infiltration of M2 macrophages, neutrophils and CD4+ T cells was observed between SPA and Non-SPA. The analysis of mapped TCGA digitized H&E-stained images also demonstrated higher proportion of tumor-infiltrating lymphocytes (TILs) and macrophages in SPA (**Figure S3B**). IHC analysis of the 103 LUADs in LUAD-NCC confirmed that SPA had higher density of CD8+ T cells (P=0.007) and macrophages (P=0.023) infiltration (**Figures 6B**, **S3E**) while no difference in M2 macrophages, CD4+ T cells, or B cells infiltration was found between SPA and Non-SPA. Th17 cell is generally associated with improved overall survival (50). In this study, using the Th17 cell signature (**Table S2**) (18), we found that SPA had lower Th17 cells infiltration as compared with Non-SPA (**Figure S3C**).

Further, we explored the correlation between pathological subtypes and the four types of tumor immune microenvironment classified based on CD8A and PD-L1 expression (51). Positive CD8A and PD-L1 were defined as expression above the median level. As expected, we observed that SPA had a higher proportion of type I tumor immune microenvironment (PD-L1+/CD8A+) than Non-SPA (**Figure 6C**), indicating adaptive immune resistance in SPA. A recent study revealed that tumors with type I tumor immune microenvironment may have more complex genomic intratumor heterogeneity (52), which was associated with postoperative recurrence and poor survival in lung cancer (53, 54). Therefore, genomic intratumor heterogeneity was compared between SPA and Non-SPA. As expected, SPA was associated with increased intratumor heterogeneity (**Figure 6D**).

### β-Catenin protein level and chemotherapeutic outcome-related signatures in SPA and Non-SPA

β-Catenin protein level was reported to inversely correlate with T cell infiltration (55). Therefore, we compared the abundance of β-Catenin protein between SPA and Non-SPA using the proteomic data in LUAD-TCGA and LUAD-NCC. We noted significantly lower levels of β-Catenin in SPA (**Figure 6E**). Finally, to explore why adjuvant chemotherapy was unsatisfactory in SPA at transcriptome level, we curated two gene expression signatures named chemoresistence signature and chemotherapy response signature (**Table S2**) (29, 30), which were associated with chemotherapeutic outcome, and calculated signature score for each patient. As expected, SPA showed significantly higher chemoresistence signature scores and lower chemotherapy response signature scores in both LUAD-TCGA and LUAD-Asia (**Figure S3D**).

### Correlation between pathological subtypes and published transcriptomic or proteomic subtypes

**Figure 7:**
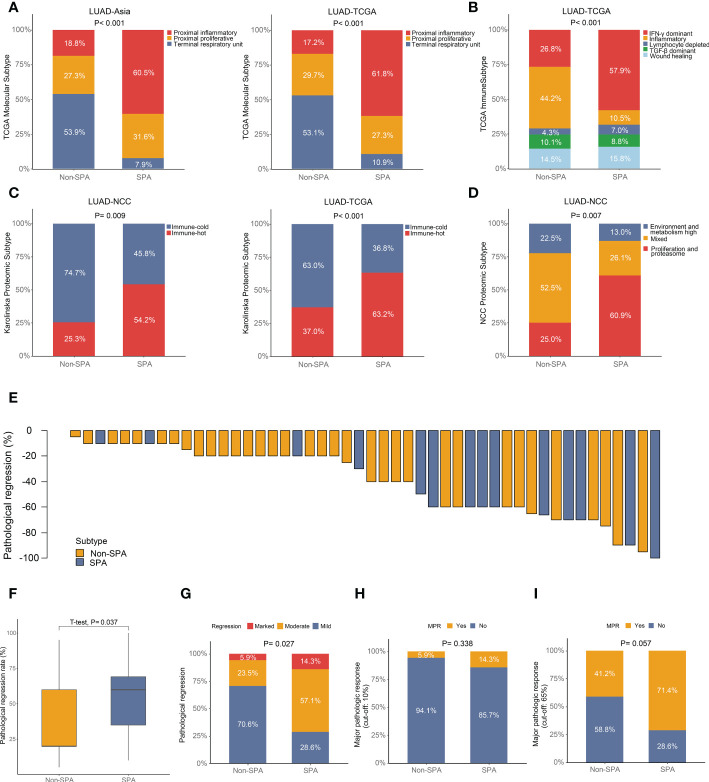
Association of published transcriptomic or proteomic subtypes with pathological subtypes and neoadjuvant immunotherapy efficacy in SPA versus Non-SPA. **(A)** Percentage of TCGA molecular subtypes in SPA and Non-SPA. **(B)** Percentage of TCGA immune subtypes in SPA and Non-SPA. **(C)** Percentage of Karolinska proteomic subtypes in SPA and Non-SPA. **(D)** Percentage of NCC proteomic subtypes in SPA and Non-SPA. **(E)** Pathological regression rate in each patient. **(F, G)** Comparison of pathological regression between SPA and Non-SPA. **(H, I)** Comparison of major pathological response between SPA and Non-SPA. SPA, solid predominant adenocarcinoma; Non-SPA, non-solid predominant adenocarcinoma; MPR, major pathological response.

To validate the aforementioned findings, we assessed the correlation between pathological subtypes (SPA versus Non-SPA) and different transcriptomic and proteomic subtypes previously published. First, we evaluated the TCGA molecular subtypes. We found that SPA was enriched for the “Proximal-inflammatory” subtype while Non-SPA was enriched for the “Terminal respiratory unit” subtype (**Figure 7A**). Next, the TCGA immune subtypes were assessed. The analysis revealed that SPA consisted primarily of the “IFN-γ dominant” subtype (**Figure 7B**), consistent with the result of unbiased GSEA which showed that SPA was enriched for the IFN-γ response pathway (**Figure S2C** and **Table S5**). Then, SPA and Non-SPA were classified into the Karolinska proteomic subtypes. Consistent with higher immune infiltration in SPA, SPA showed enrichment of the “Immune-hot” subtype in both LUAD-TCGA and LUAD-NCC (**Figure 7C**). Finally, analysis of the proteomic subtypes recently proposed by our group (NCC proteomic subtypes) showed that SPA was enriched for the “Proliferation and proteasome” subtype (**Figure 7D**).

### Validation of the better suitability of immunotherapy for SPA

The aforementioned multi-omics analyses revealed that SPA was more suitable for immunotherapy as compared with Non-SPA. To validate this finding, we investigated the non-small cell lung cancer patients who received neoadjuvant immunotherapy (alone or in combination with chemotherapy or angiogenesis inhibitor) from October 2018 to May 2022 in our center, of which 48 LUAD patients with detailed pathological results were included for further analysis (**Table S6**). We first compared the pathological regression rates between these two subtypes. As expected, SPA had significantly higher regression rates than Non-SPA (**Figures 7E, F**). Next, we stratified the pathological regression rates into three groups: marked regression (regression rate: 80-100%), moderate regression (regression rate: 40-80%), and mild regression (regression rate: 0-40%). Patients with marked regression were enriched in SPA while patients with mild regression were enriched in Non-SPA (**Figure 7G**). Further, we compared the major pathological response (MPR) between these two subtypes. When the cut-off value was set as ≦10% viable malignant cells, SPA had a higher proportion of MPR (14.3% versus 5.9%) than Non-SPA although the difference was not significant (**Figure 7H**). When using the newly proposed cut-off value of MPR for LUAD (≦65% viable malignant cells) (56), patients with MPR were significantly enriched in SPA (**Figure 7I**).

## Discussion

The newly proposed LUAD subtypes have been reported to be associated with patient prognosis and therapeutic response (1–5). Consistent with previous findings (2, 5), our study confirmed that SPA was an independent predictor of early recurrence and poor survival. To explore the molecular mechanisms underlying the unfavorable outcomes and differential therapeutic responses of SPA, we conducted a comprehensive multi-omics analysis which offered a multifaceted view of SPA and led to the optimization of therapy for SPA. Striking differences in genomic, transcriptomic and immune landscape were found between SPA and Non-SPA, which were quite conserved across different ethnic backgrounds.

Higher frequency of TP53 mutation and lower frequency of EGFR mutation were observed in SPA. TP53 plays an important role in maintaining genomic stability and its mutation can induce genomic instability and enhance tumor cell proliferation (45, 47). Our recent study revealed that the coexistence of TP53 mutation was associated with inferior survival in EGFR-mutated LUAD (14). Here, we confirmed this finding in a larger patient population. Importantly, in this study, for the first time, we found that EGFR/TP53 co-mutation was enriched in SPA. SPA was reported to be associated with poor response to EGFR-TKIs in EGFR-mutated LUAD (4) while the underlying molecular mechanism remains unknown. EGFR T790M secondary mutation, and MET amplification are two possible mechanisms of EGFR-TKIs resistance (57, 58). However, in this study, no such alternation was found in SPA with EGFR mutations. Recently, EGFR/TP53 co-mutation was found to be related to resistance to EGFR-TKIs (59). Therefore, it can be postulated that the enrichment of EGFR/TP53 co-mutation in SPA may play a critical role in its poor response to EGFR-TKIs. Additionally, we found that SPA had significantly lower frequency of therapeutically targetable alterations despite more driver mutations in this subtype. Collectively, targeted therapy may be less suitable for SPA.

Immunotherapy has renovated the standard treatment for lung cancer. However, durable response occurs only in a small subset of patients (10, 11). Thus, it is imperative to identify patients who could benefit from immunotherapy. Genomic profiling revealed that SPA had stronger immunogenicity and was enriched for genomic-associated biomarkers for immunotherapy (TMB, neoantigen load, T cell receptor; SWI/SNF complex, POLE, PTPRD, EPHA7 and NOTCH mutation; KRAS/TP53 and DDR co-mutations; TRAF2 loss; smoking and APOBEC signature), indicating potential for immunotherapy. Higher TMB and neoantigen load were observed in SPA, suggesting greater immunogenicity and a stronger immune response in this subtype (11). Antigen-specific T cell receptor is critical for recognition of neoantigens. The diversity of the T cell receptor repertoire has been reported to be associated with response to immunotherapy (60). Here, we found SPA had greater T cell receptor diversity compared with Non-SPA. The SWI/SNF complex (SMARCA4, ARID1A, ARID2, ARID1B, PBRM1, and SMARCB1) plays an important role in chromatin maintenance and genomic abnormalities of the complex may induce genomic instability, higher TMB, and aggressive biological behavior (61). Recent evidence suggested that SWI/SNF-mutant lung cancer was associated with improved efficacy of immunotherapy (38). Three of the SWI/SWF genes (SMARCA4, ARID1A, ARID2) were found to be mutated more frequently in SPA. Mutations in these genes have also been shown to predict better clinical outcomes in immunotherapy (37, 38). PTPRD and EPHA7 mutation are two new biomarkers for predicting the efficacy of immunotherapy independent of TMB or PD-L1 expression in lung cancer (41, 42). Both of them occurred more frequently in SPA in this study. Deleterious NOTCH mutation was also identified as a predictor to efficacious immunotherapy independent of TMB (19). Here, we found that deleterious NOTCH1/2/3 mutations were more common in SPA (**Figure 3G**). KRAS/TP53 co-mutation is an established predictive factor in guiding immunotherapy in lung cancer, which is associated with increased PD-L1 expression and T cell infiltration as well as augmented tumor immunogenicity (45). SPA showed significantly higher frequency of KRAS/TP53 co-mutation than did Non-SPA. Additionally, tobacco and APOBEC signature and co-mutations in DDR pathways were also reported to be associated with immunotherapy efficacy (44, 46) and all of them were enriched in SPA in this study. Overall, we demonstrated that SPA might be a potential subgroup suitable for immunotherapy at genome level.

Similar to genomic profiling, transcriptome analyses also supported SPA as a potential subgroup for immunotherapy, with significantly increased expression of signatures related to response to immunotherapy (27, 31, 32) (CD8 T effector, immune checkpoint, IFNγ-6, chemokine, and APM signature and GEP score) and an inflamed tumor immune microenvironment characterized by higher infiltration of TILs (CD8+T cells, M1 macrophages, Th1 and Th2 cells). Studies have shown that higher TIL infiltration is associated with better response and improved clinical outcomes in patients who receive immunotherapy (32, 62).

In this study, we found that consistent with the aggressive clinicopathologic characteristics of SPA (lower degree of differentiation, higher rate of lymph node metastasis, and higher pathological stage), SPA was associated with significantly lower TTF-1 and Napsin-A expression as well as higher proliferation score and Ki67 expression. Here, we observed the paradoxical coexistence of high TIL infiltration and tumor progression in SPA. One explanation is that the high proliferation rate may override the immune response in SPA. Another hypothesis is that SPA has already been remodeled by the type I tumor immune microenvironment (characterized by adaptive immune resistance) (51) and has escaped immune recognition. In this study, we observed that SPA was enriched for type I tumor immune microenvironment (PD-L1+/CD8A+) as compared with Non-SPA. T cells exposed to persistent antigen stimulation can become ‘exhausted’, lose robust effector functions, upregulate multiple immune checkpoints and fail to inhibit tumor progression (63). We found that several critical genes related to exhausted T cells, including TIM-3, TIGIT, PD-1, LAG3, and CXCL13 (63, 64), were all upregulated in SPA (**Figure 5A**), indicating an ongoing ‘exhausted’ antitumor immune response in this subtype. It has been reported that hypoxia could lead to the exhaustion of T cells and induce immunosuppression (65, 66). In this study, GSEA revealed that hypoxia-related pathway was enriched in SPA. Higher hypoxia score and increased HIF-1α expression were observed in SPA. The rapid growth of SPA with high expression of Ki67 might also be correlated to T cell exhaustion (64). Immunotherapy can reverse the exhaustion status of T cells and release an effective host antitumor immune response (10). Studies have shown that increased expression of T cell exhaustion markers is an indicator of good response to immunotherapy (62, 67). Additionally, recent evidence suggested that HIF-1α inhibition was able to alleviate tumor immunosuppression induced by hypoxia and sensitize tumor’s response to immunotherapy in lung cancer (65). Collectively, at transcriptome level, we demonstrated that SPA was more aggressive while prone to respond to immunotherapy especially when it is combined with HIF-1α inhibition

Before the era of immunotherapy, adjuvant chemotherapy used to be the standard treatment after resection although the efficacy was barely satisfactory in SPA (3, 5). Studies have shown that overexpression of PD-L1 and HIF-1α, hypoxic microenvironment, and TP53 mutation are associated with poor response to chemotherapy (68–71). All these features were enriched in SPA. In addition, SPA had significantly higher chemoresistence signature scores and lower chemotherapy response signature scores, indicating poor response to chemotherapy. Therefore, chemotherapy seemed less suitable for SPA.

To validate the aforementioned findings, we assessed the correlation between pathological subtypes (SPA versus Non-SPA) and different transcriptomic and proteomic subtypes previously published. SPA consisted primarily of the “Proximal-inflammatory” molecular subtype, the “IFN-γ dominant” immune subtype, the “Immune-hot” and “Proliferation and proteasome” proteomic subtype. All the transcriptomic and proteomic subtypes enriched in SPA had characteristics similar to those identified within SPA in this study: 1) the “Proximal-inflammatory” molecular subtype had high frequency of TP53 mutation and NF1/TP53 co-mutation, high mutation burden and more immune cell infiltration (12, 72); 2) the “IFN-γ dominant” immune subtype was characterized by high proliferation rate and intratumor heterogeneity, high TIL infiltration and M1/M2 macrophage polarization, elevated expression of CD8 T cell-associated signature and poor survival (18); 3) the “Immune-hot” proteomic subtype was associated with high TMB, elevated PD-L1 and CXCL9 expression, high T-cell infiltration, proficient antigen presentation and activated IFN-γ signaling (25); 4) the “Proliferation and proteasome” proteomic subtype was characterized by poor differentiation, low expression of TTF-1 and Napsin-A, high TMB and unfavorable prognosis (14).

Finally, to further confirm the finding that immunotherapy was more suitable for SPA than for Non-SPA based on the multi-omics analyses, we investigated a cohort of LUAD patients who received neoadjuvant immunotherapy. We found that SPA had higher pathological regression rates than Non-SPA and that patients with MPR were enriched in SPA, confirming that SPA was more prone to respond to immunotherapy.

Several limitations of this study should be acknowledged. First, this study was mainly based on the retrospective profiling of genomic and transcriptomic data. However, this limitation could be greatly minimized by the large population size and the result consistency across ethnically distinct cohorts. Prospective studies are still required to confirm the distinct genomic and immune landscape in SPA. Second, due to the immature survival data, we used pathological response as a surrogate to indicate response to neoadjuvant immunotherapy. Overall survival should be examined to further confirm the robustness of our findings when the survival data become mature. Third, in this study, our analyses were based on the predominant histologic subtype of LUAD, yet minor components of other subtypes might have confounding impacts. Further studies based on single-cell sequencing are needed to depict a more precise and comprehensive genomic and immune landscape of SPA.

In conclusion, we conducted a comprehensive analysis of the genomic, transcriptomic, and immune landscape of SPA and Non-SPA, unraveling the underlying mechanisms of the differential prognosis and therapeutic response between these two subtypes. Notably, our analysis indicated that compared with Non-SPA, SPA was more suitable for immunotherapy while less suitable for chemotherapy and targeted therapy. Our study enabled a greater understanding of the molecular mechanisms underlying the differential clinical behaviors of LUAD histologic subtypes and paved the way for tailoring bespoke treatments for LUAD patients.

## Data availability statement

The raw RNA sequencing data of LUAD-NCC presented in the study are deposited in the GEO repository, accession number GSE140343; the raw proteomic data of LUAD-NCC presented in the study are deposited in the iProx Consortium database, accession number IPX0001804000; as publicly sharing of the raw genomic data is restricted by the regulation of the Human Genetic Resources Administration of China, detailed results of whole exome sequencing of LUAD-NCC are available from the supplementary materials of our previously published study (https://doi.org/10.1016/j.cell.2020.05.043). The clinicopathologic, genomic, transcriptomic, and proteomic data of LUAD-TCGA can be retrieved from the TCGA data portal (https://portal.gdc.cancer.gov/projects/TCGA-LUAD). For LUAD-MSKCC, the clinicopathologic and genomic data can be retrieved from the cBioPortal database (https://www.cbioportal.org/study/summary?id=luad_mskcc_2020). The clinicopathologic, genomic, and transcriptomic data of LUAD-Singapore can be retrieved from the Singapore Oncology Data Portal (https://src.gisapps.org/OncoSG/). All other data supporting the findings of this study are available from the corresponding author upon reasonable request.

## Ethics statement

The studies involving human participants were reviewed and approved by the Ethics Committee of National Cancer Center/Cancer Hospital, Chinese Academy of Medical Sciences and Peking Union Medical College (Approval No. 2016YJC-01). The patients/participants provided their written informed consent to participate in this study.

## Author contributions

FL, TX, and YM conceived and designed the study. FL, SW, and YW contributed to the sample collection and patient data management. FL, SW, YW, ZL, DJ, HY, LF, and SZ performed analysis and interpretation of data. FL, TX, and YM wrote the manuscript. All authors contributed to the article and approved the submitted version.
