# Supplementary material for: Multi-omics analysis unravels the underlying mechanisms of poor prognosis and differential therapeutic responses of solid predominant lung adenocarcinoma
Source: Front Immunol. 2023 Feb 9;14:1101649. doi: 10.3389/fimmu.2023.1101649 (PMC9946976; doi:10.3389/fimmu.2023.1101649)
Supplement: Supplementary file 2 [file Image_1.pdf]

Supplementary Figures

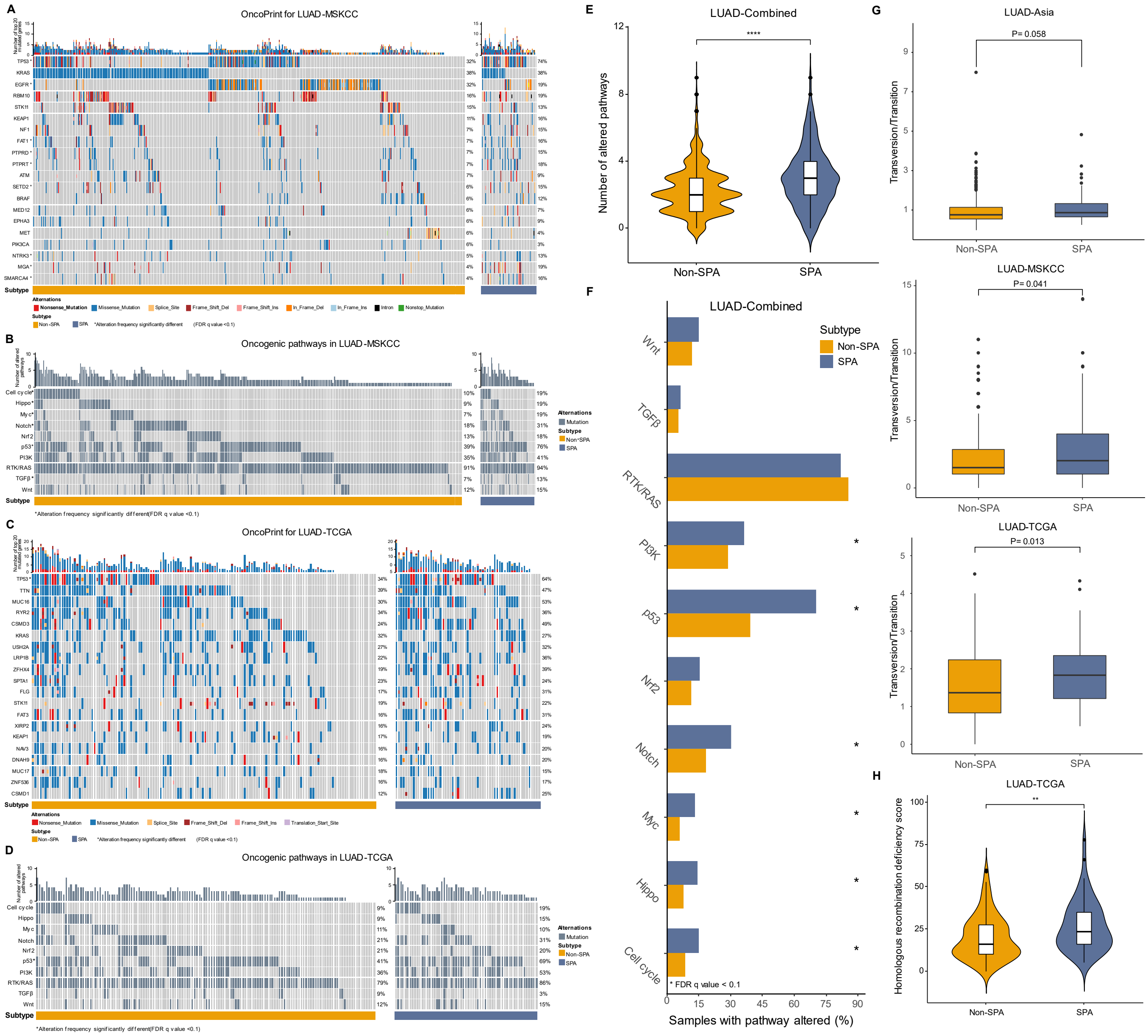

Figure S1. Genomic landscape of SPA and Non-SPA. (A and C) OncoPrint of the top 20 mutated genes in LUAD-MSKCC and LUAD-TCGA. *FDR q value < 0.1 **(B and D)** OncoPrint of the 10 hallmark oncogenic pathways in LUAD-MSKCC and LUAD-TCGA. *FDR q value < 0.1 **(E)** Number of altered pathways in SPA and Non-SPA. ****P < 0.0001 **(F)** Mutation frequency of 10 hallmark oncogenic pathways in SPA and Non-SPA in LUAD-Combined. *FDR q value < 0.1 **(G)** Transversion/transition rate in SPA and Non-SPA across

the three cohorts. **(H)** Comparison of homologous recombination deficiency scores between SPA and Non-SPA. **P < 0.01 Abbreviations: SPA, solid predominant adenocarcinoma; Non-SPA, non-solid predominant adenocarcinoma.

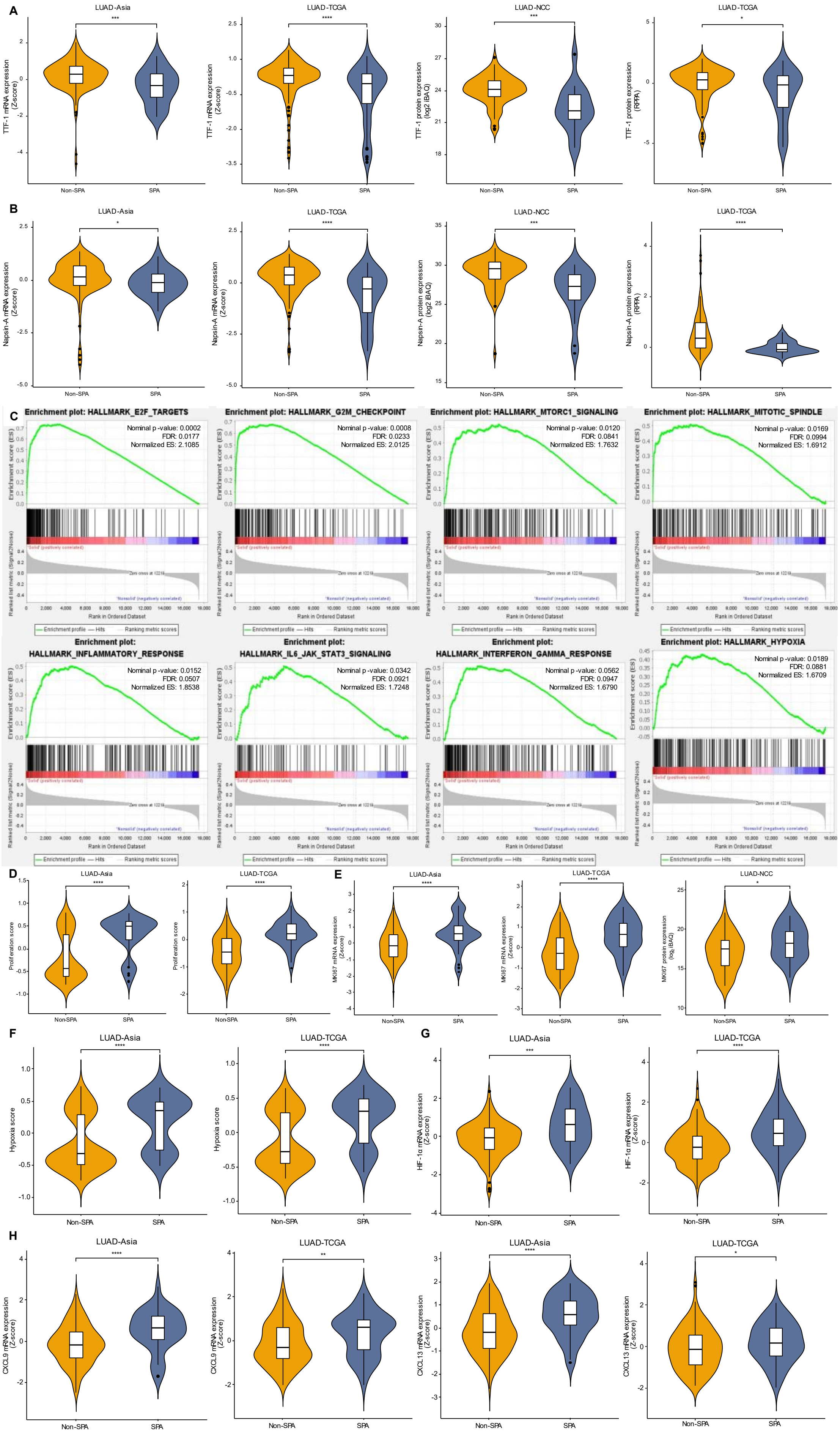

A
LUAD-Asia
LUAD-TCGA
LUAD-NCC
LUAD-TCGA
TTF-1 mRNA expression (Z-score)
TTF-1 protein expression (log2 iBAQ)
TTF-1 protein expression (RPPA)
Non-SPA
SPA
B
Napsin-A mRNA expression (Z-score)
Napsin-A protein expression (log2 iBAQ)
Napsin-A protein expression (RPPA)
C
Enrichment plot: HALLMARK_E2F_TARGETS
Nominal p-value: 0.0002
FDR: 0.0177
Normalized ES: 2.1085
Enrichment plot: HALLMARK_G2M_CHECKPOINT
Nominal p-value: 0.0008
FDR: 0.0233
Normalized ES: 2.0125
Enrichment plot: HALLMARK_MTORC1_SIGNALING
Nominal p-value: 0.0120
FDR: 0.0841
Normalized ES: 1.7632
Enrichment plot: HALLMARK_MITOTIC_SPINDLE
Nominal p-value: 0.0169
FDR: 0.0994
Normalized ES: 1.6912
Enrichment plot: HALLMARK_INFLAMMATORY_RESPONSE
Nominal p-value: 0.0152
FDR: 0.0507
Normalized ES: 1.8538
Enrichment plot: HALLMARK_IL6_JAK_STAT3_SIGNALING
Nominal p-value: 0.0342
FDR: 0.0921
Normalized ES: 1.7248
Enrichment plot: HALLMARK_INTERFERON_GAMMA_RESPONSE
Nominal p-value: 0.0562
FDR: 0.0947
Normalized ES: 1.6790
Enrichment plot: HALLMARK_HYPOXIA
Nominal p-value: 0.0189
FDR: 0.0881
Normalized ES: 1.6709
Enrichment score (ES)
Ranked list metric (Signal2Noise)
Rank in Ordered Dataset
Enrichment profile — Hits Ranking metric scores
D
Proliferation score
E
MKI67 mRNA expression (Z-score)
MKI67 protein expression (log2 iBAQ)
F
Hypoxia score
G
HIF-1α mRNA expression (Z-score)
H
CXCL9 mRNA expression (Z-score)
CXCL13 mRNA expression (Z-score)

Figure S2. Comparison of transcriptomic profiles between SPA and Non-SPA and gene set enrichment analysis of SPA versus Non-SPA. (A and B) Comparison of mRNA expression and protein abundance of TTF-1 and Napsin-A between SPA and Non-SPA in LUAD-Asia and LUAD-TCGA. **(C)** Significantly enriched hallmark pathways in SPA. **(D)** Proliferation scores in SPA and Non-SPA in LUAD-Asia and LUAD-TCGA. **(E)** Comparison of mRNA expression and protein abundance of MKI67 between SPA and Non-SPA. **(F)** Hypoxia scores in SPA and Non-SPA in LUAD-Asia and LUAD-TCGA. **(G)** Comparison of mRNA expression of HIF-1α between SPA and Non-SPA. **(H)** Comparison of mRNA expression of CXCL9 and CXCL13 between SPA and Non-SPA. SPA, solid predominant adenocarcinoma; Non-SPA, non-solid predominant adenocarcinoma; ****$P < 0.0001$, ***$P < 0.001$, **$P < 0.01$, *$P < 0.05$.

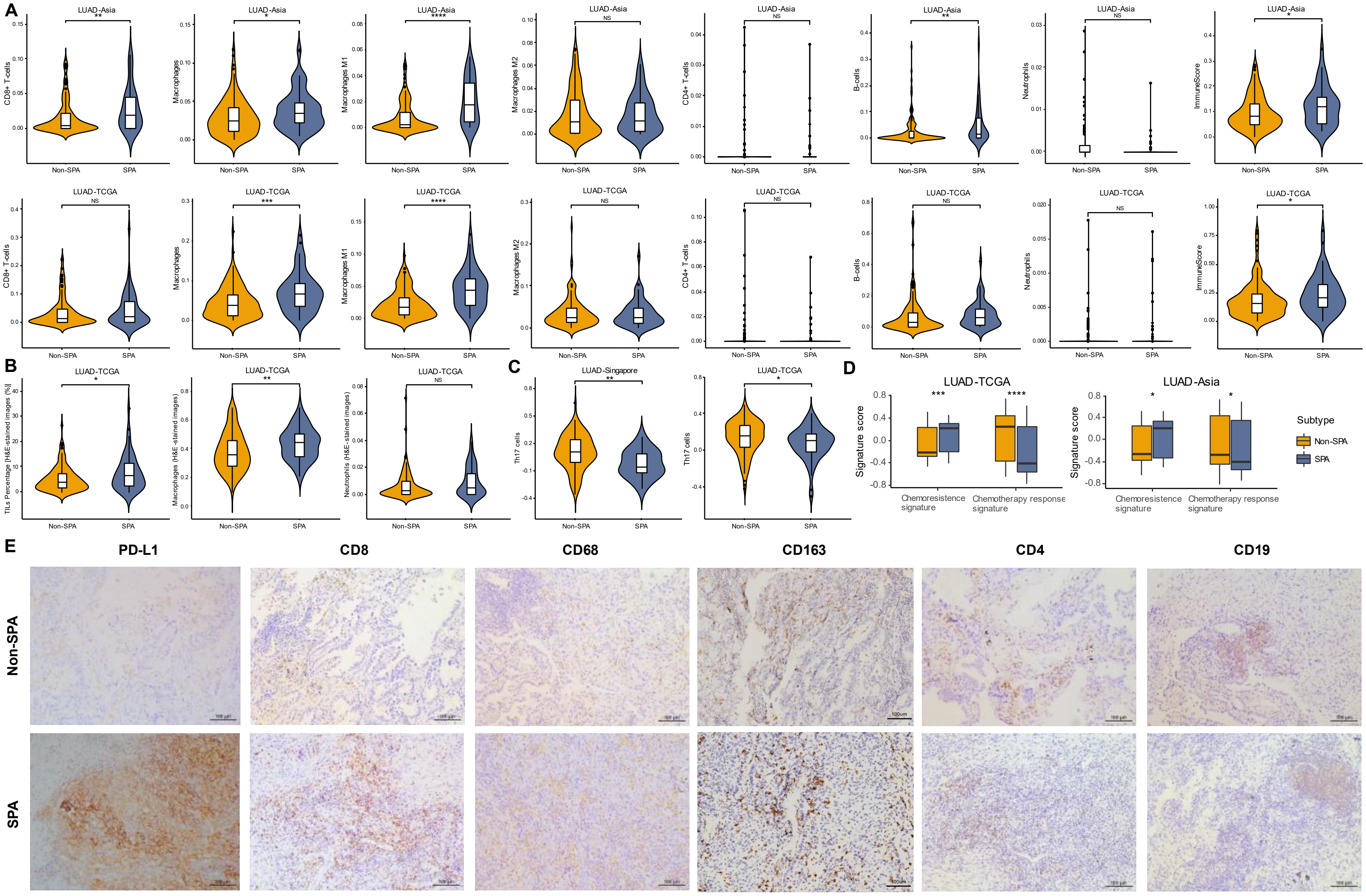
A
LUAD-Asia
CD8+ T-cells
Macrophages
Macrophages M1
Macrophages M2
CD4+ T-cells
B-cells
Neutrophils
ImmuneScore
Non-SPA
SPA
NS
LUAD-TCGA
B
TILs Percentage [H&E-stained images (%)]
Macrophages (H&E-stained images)
Neutrophils (H&E-stained images)
C
LUAD-Singapore
Th17 cells
D
LUAD-TCGA
LUAD-Asia
Signature score
Chemoresistence signature
Chemotherapy response signature
Subtype
Non-SPA
SPA
E
PD-L1
CD8
CD68
CD163
CD4
CD19
Non-SPA
SPA
100um

Figure S3. Immune cell infiltration in SPA and Non-SPA. (A) Comparison of the infiltrating immune cells estimated by xCell between SPA and Non-SPA in LUAD-Asia and LUAD-TCGA. **(B)** The spatial fractions of tumor regions with TILs, macrophages and neutrophils estimated by analysis of mapped TCGA digitized H&E-stained slides in SPA and Non-SPA. **(C)** Comparison of Th17 cell infiltration between SPA and Non-SPA in LUAD-Asia and LUAD-TCGA. **(D)** Comparison of chemoresistence signature scores and chemotherapy response signature scores between SPA and Non-SPA. **(E)** Representative images of CD8, CD68, CD163, CD4 and CD19 immunostaining in SPA and Non-SPA form LUAD-NCC. SPA, solid predominant adenocarcinoma; Non-SPA, non-solid predominant adenocarcinoma; TIL, tumor-infiltrating lymphocyte; H&E, hematoxylin-eosin. ****$P < 0.0001$, ***$P < 0.001$, **$P < 0.01$, *$P < 0.05$; NS, not significant.
